# Synthesis and Bio-Imaging Application of Highly Luminescent Mercaptosuccinic Acid-Coated CdTe Nanocrystals

**DOI:** 10.1371/journal.pone.0002222

**Published:** 2008-05-21

**Authors:** Erbo Ying, Dan Li, Shaojun Guo, Shaojun Dong, Jin Wang

**Affiliations:** 1 State Key Laboratory of Electroanalytical Chemistry Changchun Institute of Applied Chemistry, Chinese Academy of Sciences, Changchun, Jilin, China; 2 Department of Chemistry, State University of New York at Stony Brook, Stony Brook, New York, United States of America; Center for Genomic Regulation, Spain

## Abstract

Here we present a facile one-pot method to prepare high-quality CdTe nanocrystals in aqueous phase. In contrast to the use of oxygen-sensitive NaHTe or H_2_Te as Te source in the current synthetic methods, we employ more stable sodium tellurite as the Te source for preparing highly luminescent CdTe nanocrystals in aqueous solution. By selecting mercaptosuccinic acid (MSA) as capping agent and providing the borate-citrate acid buffering solution, CdTe nanocrystals with high quantum yield (QY >70% at pH range 5.0–8.0) can be conveniently prepared by this method. The influence of parameters such as the pH value of the precursor solution and the molar ratio of Cd^2+^ to Na_2_TeO_3_ on the QY of CdTe nanocrystals was systematically investigated in our experiments. Under optimal conditions, the QY of CdTe nanocrystals is even high up to 83%. The biological application of luminescent MSA-CdTe to HEK 293 cell imaging was also illustrated.

## Introduction

Semiconductor nanocrystals (often known as quantum dots) have been attracting wide interest due to their various potential applications such as in optoeletronic devices [1], photovoltaic devices [2], and biological fluorescence labeling [3]. Although biological fluorescence labeling with organic dye molecules has been the focus of intensive research for visualizing the cellular structure, studying the dynamic cellular processes and even tracking the path of single molecules in cells [4], [5]. However, the intrinsic photophysical properties of organic fluorophores, which generally have broad absorption/emission profiles and low photobleaching thresholds, have limited their effectiveness in long-term imaging and multiplexing without complex instrumentation and processing [6]. Quantum dots (QDs) developed recently as a new class of fluorescent probes have sparked intense excitement in the fields of biology and medicine, because they have shown higher sensitivity and better photostability and chemical stability than conventional fluorophore markers [7]–[10]. In addition, luminescent QDs are ideal fluorophores for multiplexed optical coding because their fluorescence emission wavelength can be continuously tuned by changing the particle size, and a single wavelength can be used for simultaneous excitation of QDs with different sizes [11]–[13]. Thus, the synthesis of QDs with “high quality” (including high photoluminescence quantum yield (PL QY), narrow size distribution) has been a hot subject to be studied today.

To date, many approaches, including organic synthesis [14]–[18] and aqueous synthesis [19]–28, have been developed to prepare luminescent QDs. Although high-quality CdTe nanocrystals (NCs) can be prepared in organic phases, they are unable to be directly used in biosystems owing to the hydrophobility of these NCs. Several methods like ligand-exchange, encapsulated into a water-soluble shell and arrested precipitation in water have been used to transfer hydrophobic CdTe NCs to aqueous solution, but the PL QYs of them are normally impaired after they are subjected to this transferring process [29]–[32]. Compared with organic synthesis, aqueous synthesis has the advantages of simplicity, reproducibility, and less toxicity. However, in most of aqueous synthesis of thiol-capped CdTe NCs, either H_2_Te (a highly toxic and flammable gas) or NaHTe (an unstable compound for its spontaneous oxidation in the presence of oxygen) is utilized as the tellurium precursor, which usually needs an inert atmosphere during the synthesis [23]–[28]. Considering the complexity caused by inert atmosphere used above, it is very necessary to employ a stable Te source as the precursor when synthesizing CdTe NCs in an aqueous solution. On the other hand, the QYs of CdTe NCs synthesized in the aqueous phase was generally low (<20%) [21], [33]. However, by properly selecting thiol stabilizers like glutathione [34], [35] or mercaptopropionic acid (MPA) [36], the QYs of CdTe NCs prepared in the aqueous solution could be improved significantly (45%∼67%). To further improve the PL QY of CdTe NCs, the post-treatment process like selective photochemical etching [33], [37], or ultrasonic irradiation [38] has been employed by several groups. For instance, Bao et al. used photochemical etching approach to produce CdTe NCs with a substantial increase in PL QY up to ∼85%, but normally more than 20 days' illumination under room light of low-pressure mercury-rare-gas discharge lamps was required to bring the fluorescence QY to this value [37]. Furthermore, several new techniques like hydrothermal treatment [23]–[28], microwave irradiation [39], [40], have been employed for rapid synthesis of high quality CdTe NCs in aqueous phase. Although the preparation of CdTe NCs can be achieved in a short time, and the QYs of them are also improved a lot than before [23]–[28], [39], [40], highly active and toxic NaHTe or H_2_Te was still used as the Te source.

In our previous communication [41], we have provided an alternative approach to prepare luminescent cystine-coated CdTe NCs with QY of 10% using sodium tellurite as the Te source. As our continuing effort to develop this approach, we systematically studied such a facile one-pot approach, and demonstrated that highly luminescent CdTe NCs can be prepared by selecting mercaptosuccinic acid (MSA) as a capping ligand. It is known that the molecular structure of MSA (pK_COOH_ are 3.30 and 4.94) combines features of both mercaptopropionic acid (MPA, pK_COOH_ = 4.32) and thioglycolic acid (TGA, pK_COOH_ = 3.53), but unlike MPA and TGA which can stabilize CdTe NCs only in alkaline aqueous solution [36]–[39], MSA can also stabilize CdTe NCs in weak acidic solution. Through optimizing the growth conditions, such as the pH of solution and the concentration of precursor solutions, the fluorescence QYs of MSA-stabilized CdTe NCs can be drastically improved, even up to 83% (Rhodamine 6G as standard) at pH = 5 without any post-treatment. Particularly, at pH = 6–8 (suitable for biological application), the maximal QYs are normally larger than 70%. In addition, we further explored the feasibility for biological application of luminescent MSA-CdTe to cell imaging.

## Results and Discussion

### Synthesis of MSA-stabilized CdTe NCs

**Figure 1:**
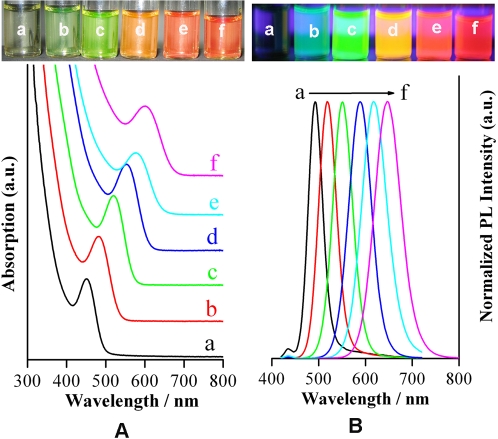
(A) The image of MSA-coated CdTe QDs with different sizes under ambient conditions (top) and the corresponding absorption spectra (bottom); (B) The image of the above-mentioned MSA-coated CdTe QDs under an ultraviolet lamp (top) and the corresponding photoluminescence spectra (bottom). The photoluminescence were at a) 493 nm, b) 519 nm, c) 551 nm, d) 589 nm, e) 617 nm, f) 647 nm. These CdTe QDs were synthesized at pH = 7.2.

At the initial stage when NaBH_4_ powder was added, the clear precursor solution would turn green in tens of seconds, depending on the pH value, the concentration of the precursor solution, and the reaction temperature. Lowering the pH value of the precursor solution or increasing the concentrations of the precursor solution or raising the reaction temperature will accelerate the formation of CdTe NCs. A large amount of bubbles were also found to release from the solution. No luminescence was observed from this crude solution owing to the very small size of the initially formed CdTe NCs. As it was heated to boiling for several minutes, the color of the solution became greener and the weak luminescence was detected. With the prolonging of reflux time, the absorption spectra of CdTe NCs as well as the PL emission spectra shifted to longer wavelengths with increasing size of the CdTe NCs as a consequence of the quantum confinement. The size of the CdTe NCs could be controlled by the duration of reflux and easily monitored by absorption and PL spectra. Figure 1 shows typical evolutions of both absorption (Figure 1A, bottom) and photoluminescence (Figure 1B, bottom) spectra of MSA-stabilized CdTe NCs prepared in aqueous solution. The images of MSA-stabilized CdTe NCs with different sizes under room light conditions (Figure 1A, top) and irradiated under an ultraviolet lamp (Figure 1B, top) are also presented. These samples were prepared in a buffer solution at pH 7.2. The spectra were measured on as-prepared CdTe colloidal solution that was taken from the refluxing reaction mixture at different interval of time. During the refluxing of about 9 h, the emission peaks of CdTe NCs shifted from 493 nm to 647 nm, and the full width at half maximum of the PL peaks increased from 35 nm to 70 nm, which indicated the narrow size distribution of the as-prepared CdTe NCs.

### Influence of pH Value, Molar Ratio and the Reaction Temperature before Heating

The influence of parameters such as the pH value of the precursor solution and the molar ratio of Cd^2+^ to Na_2_TeO_3_ on the QYs of CdTe NCs was systematically investigated in our experiments. Experimental results showed that the precursors' concentrations and pH value significantly affected the optical properties of CdTe NCs, especially the PL QYs, but the reaction temperature before heating has little effect on the PL QYs.

It is noted that the use of a suitable buffer solution is very crucial for a successful synthesis of highly luminescent CdTe NCs. During the formation of CdTe NCs, the following chemical reactions take place [41]:
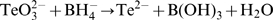
(1)

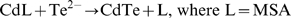
(2)


As a matter of fact, the real reaction processes are rather complicated than what the above describes. Since Te^2−^ is very sensitive to the oxygen, and we did not preclude the oxygen dissolved in the water as other workers did when NaHTe or H_2_Te was used as Te source [21], [33]. We supposed that the initially formed Te^2−^ may be reoxidized to higher valence of tellurium by the oxygen dissolved in the water but instantly reduced back to Te^2−^ by an excess NaBH_4_ in the water till the oxygen near Te^2−^ monomers was exhausted. In this regard, NaBH_4_ serves not only as a strong reductant but also produces an inert atmosphere to avoid reoxidizing Te^2−^. To ensure that all the TeO_3_^2−^ can be reduced to Te^2−^, an excess NaBH_4_ (the molar ratio of NaBH_4_/Cd^2+^ = 10/1) is always adopted. Most of the excess NaBH_4_ will be hydrolyzed or oxidized by the oxygen diffused from the air through the following reactions:
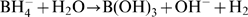
(3)

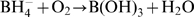
(4)


As seen from the reactions (1), (3) and (4), a large amount of B(OH)_3_ (weak acid) or OH^−^ will be produced. If the buffer capacity of the solution is low, for example, by directly adjusting the pH of the pure precursor solution (i.e., without buffer reagents) with NaOH or HCl, the pH of the solution cannot be well controlled due to the introduction of excess NaBH_4_. Indeed, the QYs of MSA-CdTe prepared by directly adjusting the pH of the precursor solution with NaOH or HCl (pH = 4–11) was typically low (<10%), and even nonluminous. Thus, it is indispensable for the reactions to take place in a buffer solution with enough buffer capacity to keep proper growth conditions of CdTe NCs during the refluxing. Under our experimental conditions, the buffer solution, which contained 15 mM borate and 15 mM citrate acid, had enough buffer capacity to withstand the changes resulting from the added NaBH_4_ powder. When the precursors like CdCl_2_, Na_2_TeO_3_ and MSA were added to this buffer solution, the pH of the solution decreased slightly (ΔpH ≈0.5). After the reactions were finished, the pH values of as-prepared CdTe nanocrystal solutions were measured to be a little higher than those of the buffer solution (ΔpH <1.2, mostly ΔpH ≈0.6).

**Figure 2:**
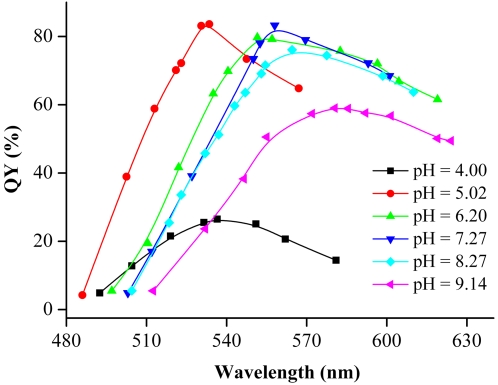
The PL QYs of CdTe NCs synthesized by using different pH precursor solutions grew at boiling temperature. Experimental conditions: 20 mg NaBH_4_ powder was added into a 50 mL of different pH buffer solutions, which contained [Cd^2+^] = 1 mM, [Na_2_TeO_3_] = 0.25 mM, and [MSA] = 3 mM.

Figure 2 shows the pH effects of the precursor solution on the QY of CdTe NCs. It can be seen that at pH = 5.0–8.0, the maximal QYs are normally larger than 70%. The pH value in our synthesis is much lower than that with TGA (pH∼11) [40] or MPA (pH >8.0) [36] as thiol stabilizers in common aqueous synthesis. For instance, when MPA was used as stabilizer for CdTe NCs, the as-prepared colloid solution would present some white precipitates if the pH value of Cd precursor solution was reduced to 6.0–7.0, which restricted the preparation of CdTe NCs in relatively lower pH aqueous solution [36]. In contrast, MSA can stabilize CdTe NCs even in weak acidic solution mostly because the structure of MSA has two carbonyl groups. Why could high-quality of CdTe NCs be obtained in weak acid solution? Previous studies of thiol complexation on the surface of growing CdS clusters indicated that in the acidic range thiols were more strongly complexed to CdS particles than to free cadmium ions [42]. Since more and more free thiols and cadmium ions will be released from the cadmium thiol complexes in the acidic range, it can be concluded that the particle surface coverage with MSA is increased when the CdTe solution becomes acidic. Therefore, more trap sites on the CdTe surface will be removed, thus leading to higher fluorescence efficiency [43]. In addition, Gao et al. thought that a thick layer of cadmium thiol complexes in the acid range was also an important factor for determining high QYs [43]. Besides the above two factors, the special structure of MSA also plays an important role in reducing the surface trap of CdTe NCs. This is because two carbonyl groups of MSA can provide better stability than other thiol compound (TGA, MPA, et al.), thus dramatically improving the fluorescence efficiency. However, further decreased pH value of the precursor solution will lead to the protonation of MSA and weaken their protecting abilities, which is unfavorable for the formation of a defect-free surface. The maximal QY of CdTe NCs prepared at pH = 4.0 is decreased to 25%.

**Figure 3:**
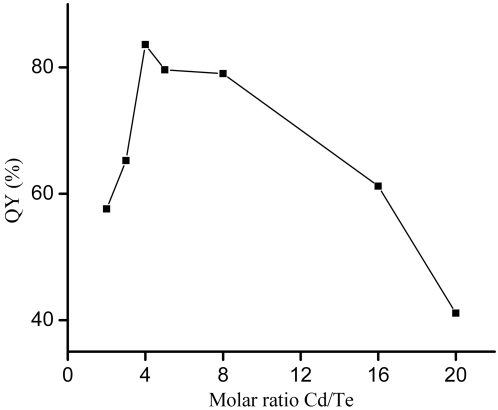
QYs of CdTe NCs prepared at different molar ratio (Cd^2+^/TeO_3_^2−^): 2.0, 3.0, 4.0, 5.0, 8.0, 16.0 and 20.0 (pH = 5.0, [Cd^2+^] = 1 mM).

The influence of the molar ratio (Cd^2+^/TeO_3_^2−^) on the QYs of CdTe NCs is shown in Figure 3. When the molar ratio of Cd^2+^/TeO_3_^2−^ is equal to 4.0, the maximal QYs of CdTe NCs is up to 83% under the condition of optimal pH value. With the increasing or decreasing in the molar ratio of Cd^2+^/TeO_3_^2−^, the maximal QYs of CdTe NCs markedly decreased. Such a trend for this influence may be related with the number of CdTe nuclei and the amount of sulfur ions doped in CdTe NCs. Once the formation of CdTe NCs was completed, the growth of the CdTe NCs during the refluxing was controlled by the Ostwald ripening process, in which smaller particles dissolve and the monomers released are consumed by the larger ones. The number of CdTe nuclei produced in the precursor solution may have an impact on the growth process of CdTe NCs. Moreover, as showed by our X-ray photoelectron spectroscopy (XPS) spectra (see Text S1, Figure S1), some sulfur ions sourced from the decomposition of Cd^2+^-thiol complex are incorporated into the CdTe NCs, which may also have an effect on the PL of CdTe NCs. A CdS shell on the surface of the CdTe NCs was known to effectively passivate the surface trap states, thus enhancing the QYs of thiol coated CdTe NCs [30], [35]. It could be reasoned that with the molar ratio of Cd^2+^/TeO_3_^2−^ = 4.0, CdTe NCs with less surface defects and proper doped amount of sulfur ions were produced, and thus the QY of them was higher than that of other samples. Other studies also emphasized the importance of the molar ratio of Cd/Te in the synthesis of high-quality CdTe QDs [34], [36].

**Figure 4:**
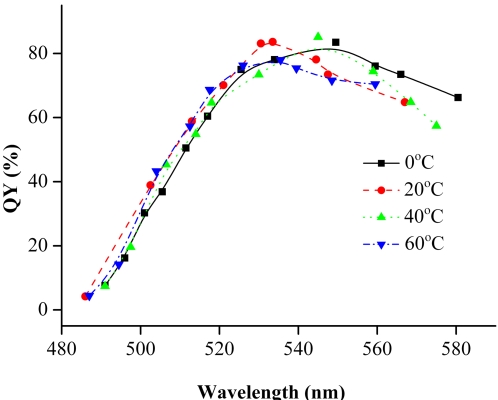
QYs of CdTe NCs prepared at different temperature before the refluxing. (Experimental conditions: pH = 5.0, [Cd^2+^] = 1 mM, MSA/Cd^2+^ = 3.0, Cd^2+^ /TeO_3_^2−^ = 4.0).

We also examine the reaction temperature of the precursor solution before the refluxing as the reducing capacity of NaBH_4_ is related not only to pH but also to the temperature. In our examined temperature range (0–60°C), no evident influence on the QYs of CdTe NCs were observed (Figure 4). This implies that the synthesis of CdTe NCs is not sensitive to the initial reaction temperature, and the growth conditions play a dominant role in the quality of CdTe NCs. Please note that the variation of temperature (precursor solution) actually influenced the PL peak position of NCs. This is because that different reaction temperature of the precursor will lead to different nucleation rate.

### TEM and XRD Characterization

**Figure 5:**
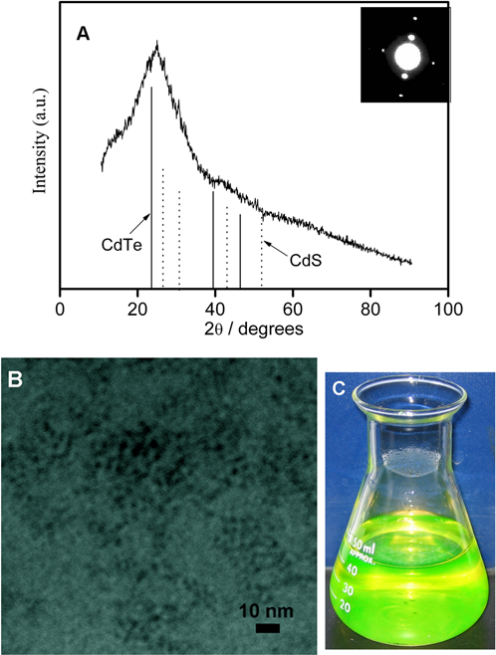
(A) XRD and SAED pattern, (B) TEM image of the MSA-capping CdTe NCs. (C) The photograph is an aqueous solution of as-prepared CdTe NCs with fluorescence QY up to 83%. It was taken under ambient room light without using additional excitation light source. The yellow-green color is from the fluorescence of CdTe NCs. (Experimental conditions: pH = 5.0, [Cd^2+^] = 1 mM, MSA/Cd^2+^ = 3.0, Cd^2+^ /TeO_3_^2−^ = 4.0).

X-ray diffraction (XRD) image and selected area electron diffraction (SAED) pattern for the MSA-capping CdTe NCs are shown in Figure 5A. The lattice parameters derived from SAED and XRD results displayed that the as-prepared NCs belonged to the cubic zinc blende structure [39]. The XRD peak positions were located between positions for a pure cubic CdTe crystal and a pure cubic CdS crystal [34]. This result indicated a partial hydrolysis of MSA in the course of refluxing and the incorporation of sulfur into CdTe NCs. XPS measurements clearly showed the presence of cadmium, tellurium, and sulfur in this sample, which further demonstrated the formation of mixed CdTe (S) QDs (see Text S1, Figure S1). A typical TEM image is shown in Figure 5B. This sample was prepared in a buffer solution at pH = 5.0 (the maximal QY of this sample is up to 83%). According to the TEM image, the CdTe NCs appear as spherical particles with the size of about 3 nm.

### Cell imaging

**Figure 6:**
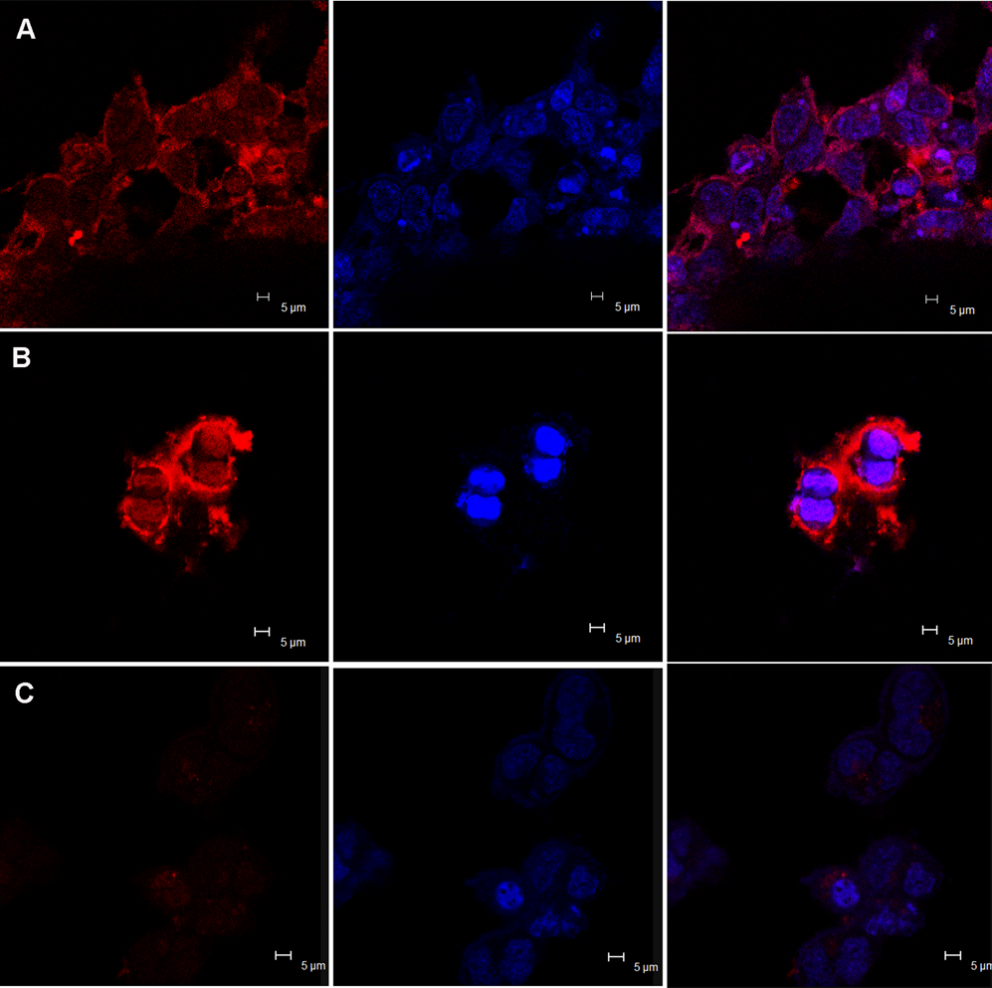
Confocal microscopic images of HEK 293 cells, treated with QD-TRF conjugate (A, B), MSA-CdTe QDs (C). (B) QDs stained the cells during cell division period. The panel to the left displays the images of CdTe QDs and their corresponding images of HEK 293 cells nuclei stained with Hoechst 33342 are shown in the middle panel. The panel to the right shows the overlays of the above two panels. Laser confocal microscopy images were obtained with laser excitation at 405 nm with a 40×objective.

To use QDs as an efficient targeted contrast agent in vitro or in vivo imaging, they need to be conjugated with specific biorecognition molecules. We choose human transferrin (TRF), an iron-transporting protein, to make QD bioconjugates. The water-soluble QDs were conjugated to transferrin using the EDC method [44]. The TRF-QDs conjugates were delivered into cells via the transferrin receptor-mediated endocytosis pathway. Cell labeling was performed with HEK 293 cells in cultures with the presence of TRF-QDs conjugates or QDs alone. Figure 6 shows the images of HEK 293 cells labeled with CdTe QDs and the cell nuclei stained by Hoechst 33342. The QDs were mainly accumulated in the cytoplasm. To validate QDs labeling specifically through the uptake of transferrin conjugation, we performed control experiment in which cells incubated with unconjugated QDs. In this control, very weak fluorescence signal was presented (Figure 6C), whereas cells labeling was positively achieved with the TRF-conjugated QDs (Figure 6A). In another experiment with the same conditions, we caught the image of cells during division process (Figure 6B), which illustrated MSA-QDs can label cells at different stages of the cell cycle, including cell division.

In conclusion, by selecting MSA as a stabilizer, we have successfully prepared high-quality CdTe NCs through such facile one-pot approach, which use an air-stable sodium tellurite as the Te source. The choice of a borate-citrate acid buffer solution as a reaction media is the key to produce high PL QY CdTe NCs. Compared with other common thiol stabilizer like TGA or MPA, MSA is somewhat unique in that its protecting ability remains even in lower pH aqueous solution (pH <8.0). As the reducing power of NaBH_4_ is pH-dependent, lower pH value can evidently enhance the reduction rate of tellurite to Te^2−^, which facilitate the formation of CdTe NCs. When TGA or MPA was chosen as stabilizer of CdTe NCs, very weak luminescent or even nonluminescent CdTe NCs were produced under similar conditions, due in great part to the requirement of higher pH reaction media (pH >8.0). Compared with current aqueous synthesis of thio-capping CdTe NCs, our approach is simpler, more reproducible and environmentally friendly. The MSA-CdTe NCs can be tuned in fluorescence from green to red and have up to 70% QY in the pH range 5.0–8.0. Moreover, the as-prepared MSA-CdTe NCs in aqueous solution were stable for months when stored in the refrigerator at 4°C, which made them more attractive for bio-imaging and biolabeling applications.

## Materials and Methods

### Materials

NaBH_4_ (98%) was from Acros. Mercaptosuccinic acid, mercaptopropionic acid, thioglycolic acid, human transferrin (≥98%), paraformaldehyde (powder, 95%), Hoechst 33342 (≥95%), N-(3-dimethylaminopropyl)-N-ethylcarbodiimide hydrochloride (EDC, ≥99%) were purchased from Sigma-Aldrich. Dulbecco's modified Eagle's medium (DMEM) was from HyClone. Trypsin was from AMRESCO. Fetal bovine serum (FBS) was from GIBCO. HEPES was from Sigma. The other chemicals were all of reagent grade. Milli-Q grade water (>18 MΩ) was used as the solvent for aqueous solutions. All chemicals were used without further purification.

### Instrumentation and measurement

UV/Vis absorption spectra were recorded by a CARY 500 UV/Vis-near-IR spectrophotometer (Varian). Fluorescence measurements were carried out at room temperature using a Perkin-Elmer LS 55 luminescence spectrometer. The room-temperature PL QY of CdTe NCs was estimated by comparison with Rhodamine 6G in absolute ethanol, the PL QY of which was assumed as 95% [39], [45].The detailed description of the measurement procedure refers to the reference 33. The excitation wavelength for recording the PL emission was 380 nm. TEM images were obtained using a JEOL-2010 electron microscope operating at 200 kV. Power X-ray diffraction (XRD) measurements were performed on Rigaku D/max 2000 x-ray diffractometer using Cu Kα radiation. For the XRD characterization, aqueous solution of as-prepared CdTe was diluted with 1 volume of ethanol and centrifuged at 4000 rpm for 10 min. The precipitate on the bottom of centrifugation tube was collected and dropped onto a glass slide and dried at room temperature. The sample for cell imaging was obtained by fixing the stained cells using 4% paraformaldehyde on a 35-mm tissue culture dish (World Precision Instruments) and acquired the fluorescence images using LEICA TCS SP2 laser scanning confocal microscope with a 40×objective.

### Synthesis of MSA-Capped CdTe QDs

CdTe QDs were synthesized according to a modified procedure developed by our group [41]. All the reactions proceeded in a buffer solution, which was composed of 15 mM Na_2_B_4_O_7_ and 15 mM citrate acid, and adjusted to different pH values with 1 M HCl or 1 M NaOH. The typical precursor solution was prepared by mixing a solution of CdCl_2_ (1 mM), Na_2_TeO_3_ (0.25 mM) and MSA (3 mM) in 50 mL of the above buffer solution (pH ∼7.0) in a one-neck flask and at room temperature (16±2°C). The molar ratio of Cd:Te:MSA was 4:1:12. After vigorously stirring for 5 min (the rate of stirring was such that the solution vortex reached the base of the stirrer), 20 mg of NaBH_4_ powder was added rapidly to the precursor solution. After the reactions proceeded for another 5 minutes, the flask was attached to a condenser and refluxed at 100°C under open-air conditions. It should be emphasized that the open-air condition is very crucial for the highly fluorescent CdTe QDs. Through controlling the reaction time, CdTe QDs with desired PL emission spectra can be obtained.

### Conjugation with the Protein Transferrin and Cell Stain

The reaction mixture was diluted with 1 volume of ethanol and centrifuged at 4000 rpm for 10 min. Redispersed the precipitation in 20 mM HEPES (pH = 7.4). QDs were conjugated to human transferrin (TRF) using the EDC coupling reaction [44]. Briefly, 200 µL of QDs solution (about 0.5 mg/mL, calculated by reference [46]) was mixed with 100 µL TRF (10 mg/mL). 0.5 mg EDC was then added to the mixture and shaken in the dark at room temperature for 2 h. Due to the TRF capping, the TRF-QDs are bigger than un-conjugated QDs. TRF-QDs and un-conjugated QDs can be separated by centrifugal filter (Millipore) with the cut off 10 kD film. After centrifugation several times, purified and concentrated TRF-QDs were obtained. The TRF-QDs bioconjugates were then incubated with HEK 293 cells (70% confluent cultured in 35-mm dishes) in DMEM supplemented with 10% fetal bovine serum at 37°C in a humidified 5% CO_2_ atmosphere. Unconjugated QDs were also incubated with HEK 293 cells and served as controls. 10 h later the cells were washed with PBS (pH = 7.4), and fixed with 4% paraformaldehyde for 20 min at room temperature. After washing three times with PBS, cells were incubated with Hoechst 33342 for 5 min at room temperature with slow shaking and then washed three times with PBS. After that, the stained cells were viewed with LEICA TCS SP2 laser scanning confocal microscope.

## Supporting Information

Text S1 (0.86 MB DOC)

Figure S1 XPS spectra of CdTe QDs prepared at pH 5.0. (A) Cd 3d; (B) Te 3d; (C) S2p.
(0.81 MB TIF)
