# Supplementary material for: Synthesis and Bio-Imaging Application of Highly Luminescent Mercaptosuccinic Acid-Coated CdTe Nanocrystals
Source: PLoS One. 2008 May 21;3(5):e2222. doi: 10.1371/journal.pone.0002222 (PMC2377334; doi:10.1371/journal.pone.0002222)
Supplement: Text S1 — (0.86 MB DOC) [file pone.0002222.s001.doc]

TEXT S1

Synthesis and Bio-Imaging Application of Highly Luminescent Mercaptosuccinic Acid-Coated CdTe Nanocrystals

Erbo Ying^1^, Dan Li^1^, Shaojun Guo^1^, Shaojun Dong^1*^, Jin Wang^2*^

*1 State Key Laboratory of Electroanalytical Chemistry Changchun Institute of Applied Chemistry, Chinese Academy of Sciences,* Changchun, Jilin, *China, 2 Department of Chemistry, State University of New York at Stony Brook, New York, United States of America*

*To whom correspondence should be addressed: [dongsj@ciac.jl.cn](mailto:dongsj@ciac.jl.cn) (SJD); [jin.wang.1@stonybrook.edu (JW)](mailto:jin.wang.1@stonybrook.edu (JW)).


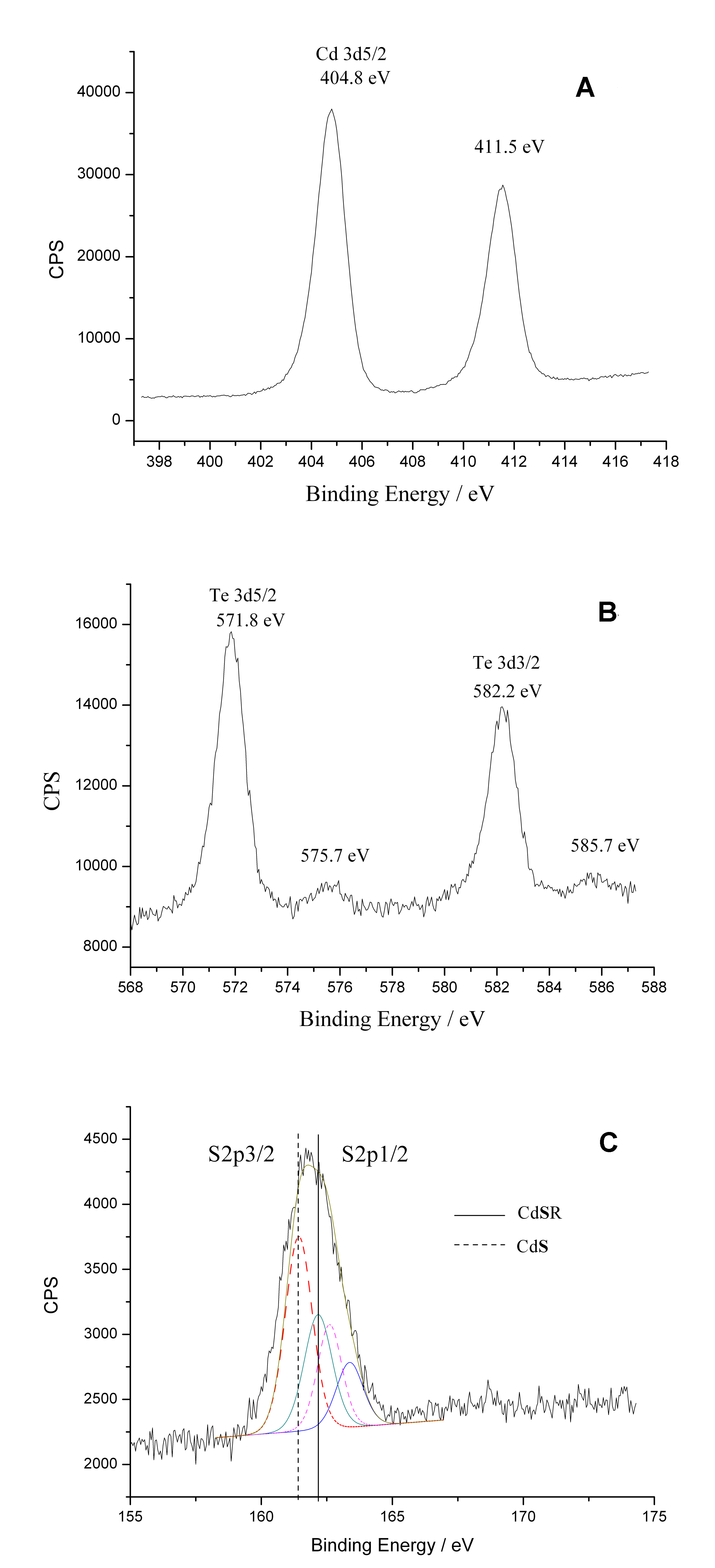


**Figure S1.** XPS spectra of CdTe QDs prepared at pH 5.0. (A) Cd 3d; (B) Te 3d; (C) S2p.
