# Supplementary figures and images for: Synthesis and Bio-Imaging Application of Highly Luminescent Mercaptosuccinic Acid-Coated CdTe Nanocrystals
Source: PLoS One. 2008 May 21;3(5):e2222. doi: 10.1371/journal.pone.0002222 (PMC2377334; doi:10.1371/journal.pone.0002222)

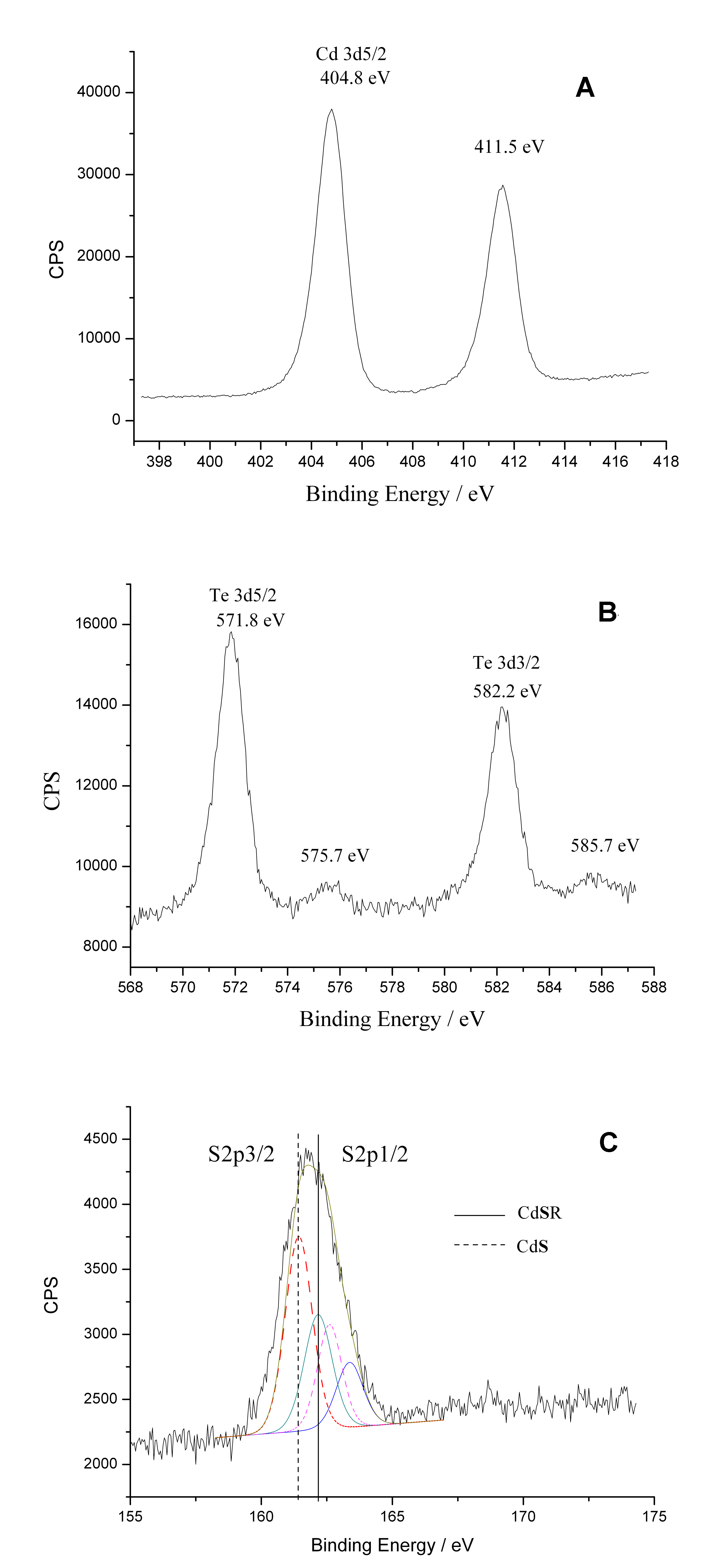

Supplement: Figure S1 — XPS spectra of CdTe QDs prepared at pH 5.0. (A) Cd 3d; (B) Te 3d; (C) S2p. (0.81 MB TIF) [file pone.0002222.s002.tif]
